# Comments on “Screening and Identification of Novel Ochratoxin A-Producing Fungi from Grapes. *Toxins* 2016, *8*, 333”—In Reporting Ochratoxin A Production from Strains of *Aspergillus*, *Penicillium* and *Talaromyces*

**DOI:** 10.3390/toxins9020065

**Published:** 2017-02-14

**Authors:** Giancarlo Perrone, Antonio F. Logrieco, Jens C. Frisvad

**Affiliations:** 1 Institute of Food Science and Production, National Reseach Council (CNR), I-70126 Bari, Italy; giancarlo.perrone@ispa.cnr.it; 2 Department of Biotechnology and Biomedicine, Technical University of Denmark, DK-2800 Kongens Lyngby, Denmark; jcf@bio.dtu.dk

**Keywords:** pure culture, correct identification, ochratoxin A, *Penicillium rubens*, *Penicillium commune*, *Talaromyces* species

## Abstract

Recently a species in the genus *Talaromyces*, a uniseriate species of *Aspergillus* section *Nigri* and an isolate each of two widespread species, *Penicillium rubens* and *P. commune*, were reported to produce ochratoxin A. This claim was based on insufficient biological and chemical data. We propose a list of criteria that need to be met before an unexpected mycotoxin producer is reported. There have only been convincing data on ochratoxin A production for *Penicillium verrucosum*, *P. nordicum*, *P. thymicola*, all from *Penicillium* series *Verrucosa*, and from species in three sections of *Aspergillus*: section *Circumdati*, section *Nigri* and section *Flavi*.

In a very recent article published online in *Toxins* (12 November 2016), Zhang and co-workers [1] screened fungal strains isolated from grapes in China, claiming they identifies “Novel Ochratoxin A (OTA)-Producing Fungi” within the genera *Aspergillus*, *Talaromyces* and *Penicillium*. In particular, they reported OTA production from species never found before to be OTA producers, namely *Talaromyces rugulosus*, *Penicillium commune*, *Penicillium rubens* and *Aspergillus aculeatus*. 

Even though it is well known that ochratoxins are produced by several fungal species belonging to the genera *Penicillium* and *Aspergillus*, no papers directly indicate that any *Talaromyces* species can produce ochratoxin [2]. On the other hand, species recently placed in the genus *Talaromyces* [3,4] have been reported to produce OTA, including *P. funiculosum* (now correctly identified as *Talaromyces funiculosus* [5,6,7]), *P. pinophilum* (now correctly identified as *T. pinophilus*) [8], *P. purpurogenum* (now correctly identified as *T. purpurogenus*) [9], *Penicillium radicum* (now correctly identified as *T. radicus*) [10], *P. rugulosum* (now correctly identified as *T. rugulosus*) [10], *P. variabile* (now correctly identified as *T. wortmanii*) [11,12,13,14,15] and *P. verruculosum* (now correctly identified as *T. verruculosus*) [9,16,17]. Several strains of all these species have been examined for production of ochratoxin A, but no isolate of the seven species of *Talaromyces* listed above was able to produce OTA [3,4,18]. 

Regarding the *Aspergillus* species producing OTA, they are distributed among the *Aspergillus* sections *Circumdati* with 20 species [19], *Nigri* with seven species [20] and *Flavi* with two species [21,22]. The uniseriate species in section *Nigri*, such as *A. aculeatus* and *A. japonicas*, have been reported to produce OTA [1,7,23,24,25,26,27,28], but among hundreds of strains properly tested, none could be confirmed to be OTA producers [21,29]. 

A large number of species of *Penicillium* have been claimed to produce OTA but we have only detected OTA in three species from the series *Verrucosa*: *P. nordicum* [30], *P. verrucosum* [30,31,32] and recently *P. thymicola* [33]. These records of OTA production by *P. verrucosum*, *P. nordicum* and *P. thymicola* have been confirmed numerous times using proper chemical characterization of OTA and proper identification of the fungi. Among other penicillia claimed to produce OTA are *P. chrysogenum*, *P. glycyrrhizacola* and *P. polonicum* from fresh or dry licorice [34] and *P. brevicompactum*, *P. crustosum*, *P. olsonii* and *P. oxalicum* isolated as endophytes in coffee [35], among many others. Again, when screening a large number of isolates of *Penicillium,* OTA has not been detected in the species listed above [2,36,37,38]. Concerning the important cereal-borne species *Penicillium polonicum*, OTA production could not be confirmed and it was suggested that OTA was not detected from this species, but rather from a contaminant in *P. polonicum*, probably *P. verrucosum* [39]. 

When claiming ochratoxin A production from a new fungal source, ideally the following measures should be taken:

1. It should be secured that the isolate is a pure culture, for example by single-spore inoculation or streaking a spore suspension on an agar medium to secure pure cultures [40].
2. Proper media and incubation conditions should be used for mycotoxin production.
3. It should be secured that the isolate is properly identified. Often ITS sequencing is not sufficient for proper identification [40,41]. The latter references can be used as a guide for proper identification of a fungal isolate.
4. The isolate should be accessioned in one or preferably two international culture collections, so the identity and purity can be checked by other scientists.
5. The presence of the toxin should be confirmed by at least three different methods, for example MS, NMR and HPLC retention time, as compared to a standard. In the case of OTA, the chlorine isotope pattern could be one way of securing that the compound is indeed OTA [32,42]. This is especially important when new records are being provided on unexpected mycotoxin production by species that have not been reported to produce them earlier.
6. The presence of biosynthetic precursors or biosynthetically related products will help confirm the presence of a mycotoxin. In the case of OTA, the presence of ochratoxin B, α or β, will help to confirm the likelihood that OTA can be produced.
7. Often phylogenetically closely related species produce a given mycotoxin, so if a mycotoxin is reported from a species unrelated to the already-known producers, extreme care should be taken to confirm that it is indeed a verified report on such a new producer.
8. Where possible and when known, check the presence and expression of gene clusters involved in the mycotoxin biosynthetic pathway.

In this respect, the paper of Zhang and co-workers [1] is flawed in that the strains claimed to produce OTA are not available for the scientific community, so it cannot be checked whether the isolates were pure cultures or whether they were indeed correctly identified. Furthermore, the analytical chemical confirmation of the actual presence of OTA in the culture is only based on HPLC-FLD (fluorescence detection), and many other fluorescent compounds with the same retention time could be mistaken for OTA. Other workers also found ochratoxin A in *P. polonicum* and *P. commune* [11,12,43,44,45,46], but Alapont et al. [44] also stated that these observations should be confirmed.

We recommend securing a more stringent review of papers where new species producing important mycotoxins are reported that are different from those species that have been repeatedly confirmed to produce the mycotoxins in question. The eight points above could be a guideline to secure that the data have been properly confirmed regarding the identity of the fungi and the toxins they produce.

## References

[1] Zhang X., Li Y., Wang H., Gu X., Zheng X., Wang Y., Diao J., Peng Y., Zhang H. (2016). Screening and identification of novel ochratoxin A-producing fungi from grapes. Toxins.

[2] Cabanes F.J., Bragulat M.R., Castellá G. (2010). Ochratoxin A production in the genus *Penicillium*. Toxins.

[3] Samson R.A., Yilmaz N., Houbraken J., Spierenburg H., Seifert K.A., Peterson S.W., Varga J., Frisvad J.C. (2011). Phylogeny and nomenclature of the genus *Talaromyces* and taxa accommodated in *Penicillium* subgenus *Biverticillium*. Stud. Mycol..

[4] Yilmaz N., Visagie C.M., Houbraken J., Frisvad J.C., Samson R.A. (2014). Polyphasic taxonomy of the genus *Talaromyces*. Stud. Mycol..

[5] Hasan H.A.H., Bagy M.M.K., Abdel-Mallek A.Y. (1993). The incidence of fungi in human axillary hair and their toxigenic potentialities. Crypt. Mycol..

[6] El-Kady I.A., Abdel-Mallek A.Y., El-Maraghy S.S.M., Hassan H.A.H. (1994). Toxigenic moulds in pesticide-treated liquid medium. Crypt. Mycol..

[7] Battilani P., Pietri A., Bertuzzi T., Languasco L., Giorni P., Kozakiewicz Z. (2003). Occurrence of ochratoxin A producing fungi in grapes grown in Italy. J. Food Prot..

[8] Battilani P., Languasco L., Pietri A., Bertuzzi T., Giorni P. Ochratoxin a in grape and wine: Causes and conditions of production. Preliminary results. Proceedings of the 5th Congress of the European Foundation for Plant pathology.

[9] Ueno Y., Kawamura O., Sugiura Y., Horiguchi K., Nakajima M., Yamamoto K., Sato S., Castagnero M., Plestina R., Dirheimer G., Chermozemsky I.N., Bartsch H. (1991). Use of monoclonal antibodies, enzyme-linked immunosorbent assay and immunoaffinity column chromatography to determine ochratoxin A in porcine sera, coffee products and toxin-prodicing fungi. Mycotoxins, Endemic Nephropathy and Urinary Tract Tumours.

[10] Torelli E., Firrao G., Locci R., Gobbi E. (2006). Ochratoxin A-producing strains of *Penicillium* spp. isolated from grapes used for production of “passito” wines. Int. J. Food. Microbiol..

[11] Mintzlaff H.-J., Ciegler A., Leistner L. (1972). Potential mycotoxin problems in mold fermented sausages. Z. Lebensm. Unters. Forsch..

[12] Ciegler A., Fennell D.I., Mintzlaff H.-J., Leistner L. (1972). Ochratoxin synthesis by *Penicillium* species. Naturwiss.

[13] Leistner L., Pitt J.I., Rodricks J.V., Hesseltine C.W., Mehlman M.A. (1977). Miscellaneous *Penicillium* toxins. Mycotoxins in Human and Animal Health.

[14] Jimenez M., Sanchis V., Mateo R., Hernandez E. (1986). *Penicillium* in pre-harvest corn in Valencia (Spain). II. Study of the enzymatic and toxigenic capacities of the species. Mycopathologia.

[15] Krivobok S., Seigle-Murandi F., Steiman R., Creppy E.E. (1995). Fungal flora and ochratoxin a production in various food and feed in France. Syst. Appl. Microbiol..

[16] Lillehoj E.B., Göransson B. (1980). Occurrence of ochratoxin- and citrinin-producing fungi on developing Danish barley grain. Acta Pathol. Microbiol. Scand. Sect. B.

[17] Bhatnagar D., Yu J.Y., Ehrlich K.C., Breitenbach M., Crameri R., Lehrer S.B. (2002). Toxins of filamentous fungi. Fungal Allergy and Pathogenicity.

[18] Frisvad J.C., Filtenborg O., Samson R.A., Stolk A.C. (1990). Chemotaxonomy of the genus *Talaromyces*. Antonie Leeuwenhoek.

[19] Visagie C.M., Varga J., Houbraken J., Meijer M., Kocsubé S., Yilmaz N., Fotedar R., Seifert K.A., Frisvad J.C., Samson R.A. (2014). Ochratoxin production and taxonomy of the yellow aspergilli (*Aspergillus* section *Circumdati*). Stud. Mycol..

[20] Varga J., Frisvad J.C., Kocsubé S., Brankovics B., Tóth B., Szigeti G., Samson R.A. (2011). New and revisited species in *Aspergillus* section *Nigri*. Stud. Mycol..

[21] Perrone G., Susca A., Cozzi G., Ehrlich K., Varga J., Frisvad J.C., Meijer M., Noonim P., Mahakarnchanakul W., Samson R.A. (2007). Biodiversity of *Aspergillus* species in some important agricultural products. Stud. Mycol..

[22] Varga J., Frisvad J.C., Samson R.A. (2011). Two new aflatoxin producing species, and an overview of *Aspergillus* section *Flavi*. Stud. Mycol..

[23] Dalcero A., Magnoli C., Hallak C., Chiacchiera S.M., Palacio G., Rosa C.A.R. (2002). Detection of ochratoxin A in animal feeds and capacity to produce this mycotoxin by *Aspergillus* section *Nigri* in Argentina. Food Addit. Contam..

[24] Battilani P., Pietri A., Giorni P., Bertuzzi T., Brabano C., Bryson R.J., Kennedy R., Magan N., Scudamore K.A. (2003). Growth and ochratoxin A production by *Aspergillus* section *Nigri* isolates from Italian grapes. Mycotoxins in food production systems.

[25] Magnoli C., Astoreca A., Ponsone L., Combina M., Palacio G., Rosa C.A.R., Dalcero A.M. (2004). Survey of mycoflora and ochratoxin A in dried vine fruits from Argentina markets. Lett. Appl. Microbiol..

[26] Varga J., Kozakiewicz Z. (2006). Ochratoxin A in grapes and grape-derived products. Trends Food Sci. Technol..

[27] Magnoli C., Astoreca A., Ponsone M.L., Fernández-Juir G.M., Berberis C., Dalcero A.M. (2007). Ochratoxin A and *Aspergillus* section *Nigri* in peanut seed at different months of storage in Córdoba, Argentina. Int. J. Food Microbiol..

[28] Ponsone M.L., Combina M., Dalcero A., Chulze S.N. (2007). Ochratoxin A and ochratoxigenic *Aspergillus* species in Argentinean wine grapes cultivated under organic and non-organic systems. Int. J. Food Microbiol..

[29] Somma S., Perrone G., Logrieco A.L. (2012). Diversity of black Aspergilli and mycotoxin risks in grape, wine and dried vine fruits. Phytopathol. Mediterr..

[30] Larsen T.O., Svendsen A., Smedsgaard J. (2001). Biochemical characterization of ochratoxin A-producing strains of the genus *Penicillium*. Appl. Environ. Microbiol..

[31] Frisvad J.C., Samson R.A., Pitt J.I. (1985). Classification of asymmetric penicillia using expressions of differentiation. Advances in Penicillium and Aspergillus Systematics.

[32] Pitt J.I. (1987). Penicillium viridicatum, Penicillium verrucosum, and production of ochratoxin A. Appl. Environ. Microbiol..

[33] Nguyen H.D., McMullin D.R., Ponomareva E., Riley R., Pomraning K.R., Baker S.E., Seifertt K.A. (2016). Ochratoxin A production by *Penicillium thymicola*. Fungal Biol..

[34] Chen A.J., Tang D., Zhou Y.Q., Da Sun B., Li X.J., Wang L.Z., Gao W.W. (2013). Identification of ochratoxin A producing fungi associated with fresh and dry liquorice. PLOS ONE.

[35] Vega F.E., Posada F., Peterson S.W., Gianfagna T.J., Chaves F. (2006). *Penicillium* species endophytic in coffee plants and ochratoxin A production. Mycologia.

[36] Frisvad J.C., Samson R.A. (2004). Polyphasic taxonomy of *Penicillium* subgenus *Penicillium*. A guide to identification of the food and air-borne terverticillate Penicillia and their mycotoxins. Stud. Mycol..

[37] Frisvad J.C., Filtenborg O. (1989). Terverticillate penicillia: Chemotaxonomy and mycotoxin production. Mycologia.

[38] Frisvad J.C., Smedsgaard J., Larsen T.O., Samson R.A. (2004). Mycotoxins, drugs and other extrolites produced by species in *Penicillium* subgenus *Penicillium*. Stud. Mycol..

[39] Mantle P., Copetti M.V., Buddie A., Frisvad J.C. (2016). Comments on “Mycobiota and Mycotoxins in Traditional Medicinal Seeds from China. *Toxins*
**2015**, *7*, 3858–3875”—In attributing ochratoxin A biosynthesis within genus *Penicilium* occurring on natural agricultural produce. Toxins.

[40] Visagie C.M., Houbraken J., Frisvad J.C., Hong S.-B., Klaassen C.H.W., Perrone G., Seifert K.A., Varga J., Yaguchi T., Samson R.A. (2014). Identification and nomenclature of the genus *Penicillium*. Stud. Mycol..

[41] Samson R.A., Visagie C.M., Houbraken J., Hong S.-B., Hubka V., Klaassen C.H.W., Perrone G., Seifert K.A., Susca A., Tanney J.B. (2014). Phylogeny, identification and nomenclature of the genus *Aspergillus*. Stud. Mycol..

[42] Frisvad J.C., Nielsen K.F., Samson R.A., Hocking A.D., Pitt J.I., Samson R.A., Thrane U. (2006). Recommendations concerning the chronic problem of misidentification of mycotoxinogenic fungi associated with foods and feeds. Advances in Food Mycology.

[43] Pohlmeier M.M., Bullerman L.B. (1978). Ochratoxin A production by *Penicillium* species isolated from cheese. J. Food Prot..

[44] Alapont C., López-Mendoza M.C., Martínez-Culebras P.V. (2014). Mycobiota and toxigenic *Penicillium* species on two Spanish dry-cured ham manufacturing planys. Food Addit. Contam. Part A.

[45] Chen A.J., Jiao X., Hu Y., Lu X., Gao W. (2015). Mycobiota and mycotoxins in traditional medicinal seeds from China. Toxins.

[46] Chen A.J., Huang L.F., Wang L.Z., Tang D., Cai F., Gao W.W. (2011). Occurrence of toxigenic fungi in ochratoxin A contaminated liquorice root. Food Addit. Contam..

